# Development and Validation of a Nomogram Prediction Model for In-hospital Mortality in Patients with Cardiac Arrest: A Retrospective Study

**DOI:** 10.31083/RCM33387

**Published:** 2025-04-10

**Authors:** Peifeng Ni, Shurui Xu, Weidong Zhang, Chenxi Wu, Gensheng Zhang, Qiao Gu, Xin Hu, Ying Zhu, Wei Hu, Mengyuan Diao

**Affiliations:** ^1^Department of Critical Care Medicine, Zhejiang University School of Medicine, 310058 Hangzhou, Zhejiang, China; ^2^Department of Critical Care Medicine, Hangzhou First People’s Hospital, Westlake University School of Medicine, 310006 Hangzhou, Zhejiang, China; ^3^Department of Critical Care Medicine, The Fourth Clinical School of Zhejiang Chinese Medicine University, 310053 Hangzhou, Zhejiang, China; ^4^Department of Critical Care Medicine, Second Affiliated Hospital, Zhejiang University School of Medicine, 310009 Hangzhou, Zhejiang, China

**Keywords:** cardiac arrest, mortality, nomogram, prediction model, LASSO regression, machine learning

## Abstract

**Background:**

Cardiac arrest (CA) is associated with high incidence and mortality rates. Hence, assessing the prognosis of CA patients is crucial for optimizing clinical treatment. This study aimed to develop and validate a clinically applicable nomogram for predicting the risk of in-hospital mortality in CA patients.

**Methods:**

We retrospectively collected the clinical data of CA patients admitted to two hospitals in Zhejiang Province between January 2018 and June 2024. These patients were randomly assigned to the training set (70%) and the internal validation set (30%). Variables of interest included demographics, comorbidities, CA-related characteristics, vital signs, and laboratory results, and the outcome was defined as in-hospital death. Variables were selected using least absolute shrinkage and selection operator (LASSO) regression, recursive feature elimination (RFE), and eXtremely Gradient Boosting (XGBoost). Meanwhile, multivariate regression analysis was used to identify independent risk factors. Subsequently, prediction models were developed in the training set and validated in the internal validation set. Receiver operating characteristic (ROC) curves were plotted and the area under these curves (AUC) was calculated to compare the discriminative ability of the models. The model with the highest performance was further validated in an independent external cohort and was subsequently represented as a nomogram for predicting the risk of in-hospital mortality in CA patients.

**Results:**

This study included 996 CA patients, with an in-hospital mortality rate of 49.9% (497/996). The LASSO regression model significantly outperformed the RFE and XGBoost models in predicting in-hospital mortality, with an AUC value of 0.81 (0.78, 0.84) in the training set and 0.85 (0.80, 0.89) in the internal validation set. The AUC values for these sets in the RFE model were 0.74 (0.70, 0.78) and 0.77 (0.72, 0.83), respectively, and those for the XGBoost model were 0.75 (0.71, 0.79) and 0.77 (0.72, 0.83), respectively. For the optimal prediction model, the AUC value of the LASSO regression model in the external validation set was 0.84 (0.78, 0.90). The LASSO regression model was represented as a nomogram incorporating several independent risk factors, namely age, hypertension, cause of arrest, initial heart rhythm, vasoactive drugs, continuous renal replacement therapy (CRRT), temperature, blood urea-nitrogen (BUN), lactate, and Sequential Organ Failure Assessment (SOFA) scores. Calibration and decision curves confirmed the predictive accuracy and clinical utility of the model.

**Conclusions:**

We developed a nomogram to predict the risk of in-hospital mortality in CA patients, using variables selected via LASSO regression. This nomogram demonstrated strong discriminative ability and clinical practicality.

## 1. Introduction

Cardiac arrest (CA) is characterized by abrupt cessation of the heart’s pumping function and is associated with a high mortality rate [1, 2]. In the United States, more than 400,000 individuals die from CA annually, with a survival-to-discharge rate of <10% [1]. Despite the widespread implementation of basic life support and substantial progress in therapeutic interventions, the management of CA remains a significant challenge [3].

Increasing the survival rate of CA survivors is a critical issue that warrants immediate attention in the field of critical care medicine. The poor prognosis of CA patients is often attributed to hypoxic-ischemic brain injury and dysfunction of other organs [4]. Prognostic assessment is an essential component of CA management. At present, serum biomarkers, neurophysiological tests, and imaging are commonly used to assess neuroprognosis for CA patients [5]. However, these methods can be influenced by sedation or limited by the unavailability of equipment and life support conditions [6, 7]. Although severity scoring systems, such as Sequential Organ Failure Assessment (SOFA), Acute Physiology and Chronic Health Evaluation (APACHE) II, and Simplified Acute Physiology Score (SAPS) II, are commonly used for prognostic assessment, their accuracy is reported to be moderate [8, 9, 10]. The advent of machine learning (ML)-based prediction models marks an innovative shift in the prognosis assessment of CA. As a specialized branch of artificial intelligence (AI), ML empowers computers to autonomously discern and learn from historical data, identifying underlying rules and patterns. By constructing precise algorithms, ML is capable of predicting targeted events or trends, thus enhancing the efficiency and accuracy of prognosis assessment [11]. However, ML-based models often suffer from the “black box” issue, that is, their internal mechanisms lack transparency, posing certain challenges in practical application [12].

A nomogram is a graphical tool that represents predictive factors in scaled segments to demonstrate the relationship between variables and outcomes [13]. Its intuitive design and ease of use allow clinicians to rapidly comprehend and apply complex prediction models [14]. Nomograms have demonstrated efficacy in predicting the incidence [15], complications [16], and outcomes [17, 18] of cardiovascular diseases. Therefore, in this study, we developed and validated a nomogram for predicting the risk of in-hospital mortality in CA patients.

## 2. Materials and Methods

### 2.1 Data Sources and Patient Cohort

This retrospective study included CA patients admitted to Hangzhou First People’s Hospital affiliated to Westlake University School of Medicine and Second Affiliated Hospital of Zhejiang University School of Medicine (Zhejiang, China) between January 1, 2018, and June 30, 2024. The inclusion criteria for CA patients were as follows: (1) survival for >24 hours after resuscitation; (2) an age of ≥18 years; and (3) availability of the records of the first intensive care unit (ICU) admission. The external validation set came from Huzhou Central Hospital and Jinhua Central Hospital. Patients were enrolled following the same inclusion and exclusion criteria. This study was approved by the Ethics Committee of Hangzhou First People’s Hospital, Westlake University School of Medicine (IIT-20230420-0077-01).

### 2.2 Data Extraction and Preprocessing

We followed the criteria developed by Riley *et al*. [19] to calculate the minimum sample size required for a binary multivariate prediction model. It was performed through the “pmsampsize” package in R (version 4.3.2, R Foundation for Statistical Computing, Vienna, Austria). The shrinkage factor, absolute difference between the apparent and adjusted Nagelkerke R2, prevalence and C-statistics were defined as 0.9, 0.05, 0.5 and 0.8, respectively. Based on these parameters, the calculated sample size was 885.

Data were extracted from the electronic medical record systems of the two hospitals. The data of 54 variables were retrieved, including demographic characteristics, comorbidities, location of arrest, presumed etiology (cardiac or non-cardiac), initial rhythm (shockable or non-shockable), treatment methods, scoring systems, vital signs, and laboratory tests within 24 hours after ICU admission. The mean values were calculated for variables with multiple measurements such as vital signs and laboratory results. The primary outcome was in-hospital mortality.

Addressing missing values was a critical step in data preprocessing. Variables with ≥25% missing data were directly excluded. For variables with <25% missing data, multiple imputation was used, introducing randomness to reflect the uncertainty of missing data more accurately than that achieved via single imputation [20]. This approach was implemented using the “mice” package in R (version 4.3.2, R Foundation for Statistical Computing, Vienna, Austria).

### 2.3 Statistical Analysis

Statistical analyses were performed using the SPSS (version 25.0, IBM Corp., Chicago, IL, USA) and R software. Continuous variables were expressed as the means with standard deviations (SDs) or medians with interquartile ranges (IQRs). Student’s *t*-test or Mann-Whitney U test was used for intergroup comparisons. Categorical variables were expressed as numbers with percentages and compared using Chi-square test or Fisher’s exact test. A *p-*value of <0.05 was considered statistically significant.

Eligible CA patients were randomly assigned to the training set (70%) and internal validation set (30%). Predictors of in-hospital mortality were identified in the training set, and three methods were compared. The Least Absolute Shrinkage and Selection Operator (LASSO) regression is a variation of the linear regression model. It incorporates a penalty function (L1 regularization term) into the general linear regression. By adjusting the lambda (λ) parameter through 10-fold cross-validation to compress some coefficients to zero, LASSO regression can achieve variable selection and model simplification [21]. Recursive Feature Elimination (RFE) is an iterative variable selection method. It repeatedly trains models and ultimately identifies the optimal subset of features with the best performance by assessing the importance of each feature and progressively removing the least important features [22]. EXtremely Gradient Boosting (XGBoost) is an ensemble learning algorithm based on tree models. It can automatically evaluate the importance of features through its internal structure. During the construction of tree models, the importance score of each feature is generated by accumulating the split contributions across all trees to provide reference for variable selection [23].

Variables selected using the three aforementioned methods were analyzed using univariate and multivariate logistic regression analyses to identify significant risk factors for in-hospital mortality in CA patients, and these factors were used to construct prediction models. Receiver operating characteristic (ROC) curves were plotted, and the area under the curves (AUC) was calculated to assess the discriminative ability of the models. Calibration curves were plotted to evaluate the consistency between predicted and actual outcomes, and decision curves were plotted to assess the practical utility of the models. The model with the highest performance was further validated in the external validation set and was subsequently represented as a nomogram for predicting the risk of in-hospital mortality in CA patients.

## 3. Results

### 3.1 Baseline Characteristics

**Fig. 1.**
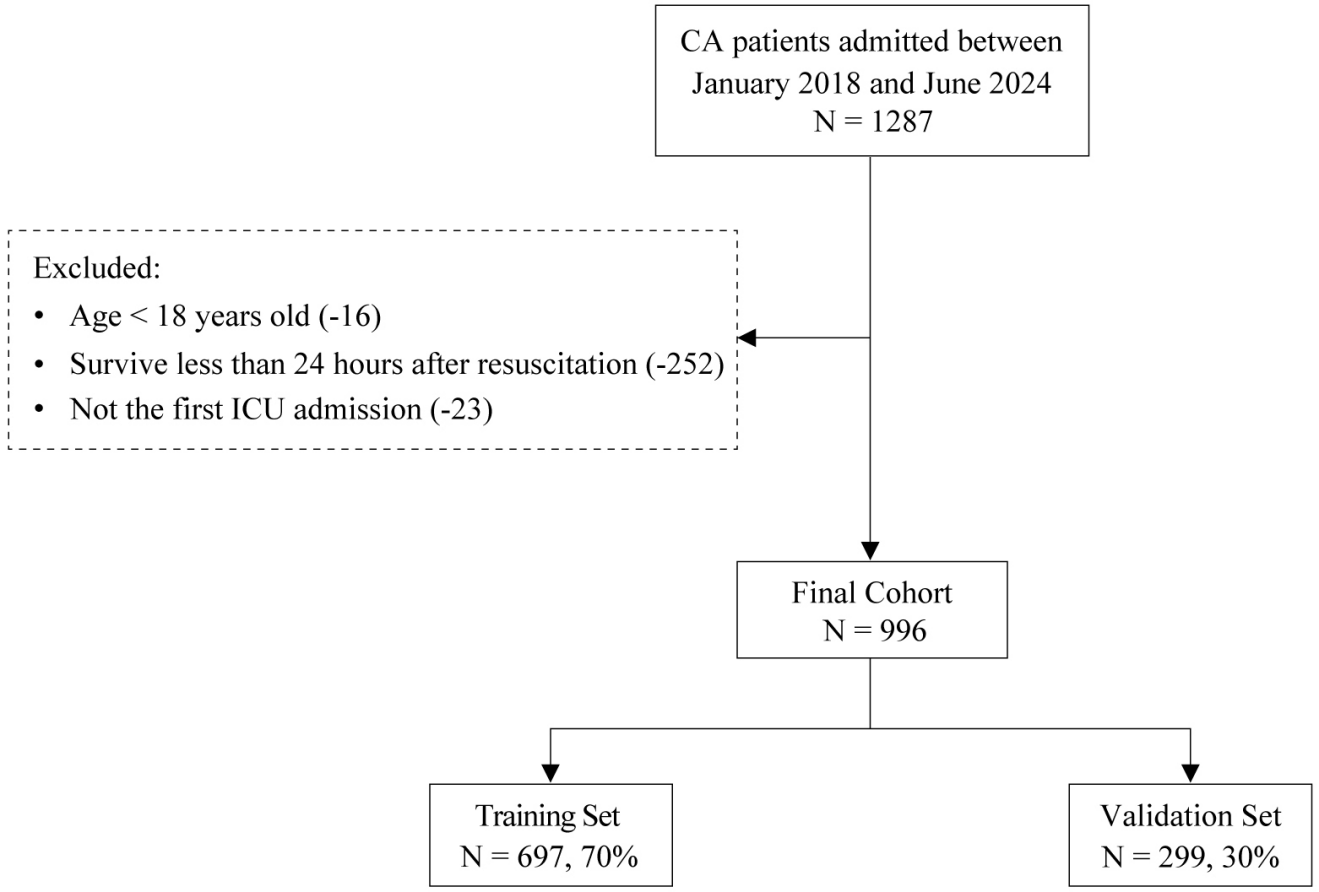
**Flowchart of patient selection**. CA, cardiac arrest; ICU, intensive care unit.

A total of 996 CA patients were included in this study (Fig. 1). Of these patients, 497 (49.9%) died upon discharge. As detailed in Table 1, age, heart rate, aspartate aminotransferase (AST), bilirubin, creatinine, blood urea nitrogen (BUN), glucose, sodium, prothrombin time (PT), international normalized ratio (INR), lactate, base excess, and SOFA scores were higher in the non-survivor group than in the survivor group. The non-survivor group had a higher prevalence of diabetes, cerebral infarction, and chronic kidney disease (CKD); more frequent use of vasoactive drugs, sodium bicarbonate, continuous renal replacement therapy (CRRT), and mechanical ventilation; a lower body temperature, hemoglobin, platelet, albumin, pH, actual bicarbonate; and less frequent use of percutaneous coronary intervention (PCI). Additionally, the survivor group had a higher ratio of cardiac cause of CA and shockable rhythms.

**Table 1.** **Baseline characteristics of participants**.

Variables		Overall (n = 996)	Survivor (n = 499)	Non-survivor (n = 497)	*p*-value
Demographic information					
	Male	675.00 (67.77)	340.00 (68.14)	335.00 (67.40)	0.805
	Age (years)	62.00 (49.00, 72.00)	61.00 (49.00, 70.00)	63.00 (50.00, 75.00)	0.002
	BMI	23.28 (20.55, 25.71)	23.40 (20.76, 25.95)	22.86 (20.50, 25.47)	0.147
Comorbidities					
	Hypertension	440.00 (44.18)	208.00 (41.68)	232.00 (46.68)	0.112
	Diabetes mellitus	194.00 (19.48)	74.00 (14.83)	120.00 (24.14)	<0.001
	Heart failure	286.00 (28.71)	147.00 (29.46)	139.00 (27.97)	0.603
	Myocardial infarction	180.00 (18.07)	101.00 (20.24)	79.00 (15.90)	0.075
	Cerebral infarction	163.00 (16.37)	68.00 (13.63)	95.00 (19.11)	0.019
	Chronic obstructive pulmonary disease	60.00 (6.02)	24.00 (4.81)	36.00 (7.24)	0.107
	Liver cirrhosis	18.00 (1.81)	10.00 (2.00)	8.00 (1.61)	0.640
	Chronic kidney disease	79.00 (7.93)	30.00 (6.01)	49.00 (9.86)	0.025
	Malignancy	103.00 (10.34)	48.00 (9.62)	55.00 (11.07)	0.453
CA-related characteristics					
	OHCA	569.00 (57.13)	286.00 (57.31)	283.00 (56.94)	0.905
	Non-cardiac cause	626.00 (62.85)	280.00 (56.11)	346.00 (69.62)	<0.001
	Shockable rhythms	274.00 (27.51)	172.00 (34.47)	102.00 (20.52)	<0.001
Treatments					
	Vasoactive drugs	873.00 (87.65)	402.00 (80.56)	471.00 (94.77)	<0.001
	Antiarrhythmic drugs	437.00 (43.88)	210.00 (42.08)	227.00 (45.67)	0.254
	Glucocorticoids	395.00 (39.66)	188.00 (37.68)	207.00 (41.65)	0.200
	Sodium bicarbonate	431.00 (43.27)	159.00 (31.86)	272.00 (54.73)	<0.001
	PCI	164.00 (16.47)	98.00 (19.64)	66.00 (13.28)	0.007
	ECMO	175.00 (17.57)	76.00 (15.23)	99.00 (19.92)	0.052
	CRRT	393.00 (39.46)	135.00 (27.05)	258.00 (51.91)	<0.001
	IABP	88.00 (8.84)	39.00 (7.82)	49.00 (9.86)	0.256
	Mechanical ventilation	966 (96.99)	471.00 (94.39)	495.00 (99.60)	<0.001
Vital signs					
	Heart rate (bpm)	90.00 (77.00, 107.00)	89.00 (75.50, 103.00)	92.00 (79.00, 111.00)	<0.001
	SBP (mmHg)	120.00 (107.00, 134.00)	121.00 (109.00, 134.50)	120.00 (105.00, 134.00)	0.133
	DBP (mmHg)	67.00 (59.00, 76.00)	68.00 (60.00, 76.00)	66.00 (57.00, 76.00)	0.055
	MBP (mmHg)	85.00 (76.00, 94.00)	86.00 (77.50, 94.00)	84.00 (75.00, 94.00)	0.053
	Respiratory rate (bpm)	16.00 (15.00, 18.00)	16.00 (15.00, 18.00)	16.00 (15.00, 19.00)	0.128
	Temperature (^∘^C)	36.50 (35.80, 37.10)	36.60 (36.00, 37.30)	36.20 (35.50, 37.00)	<0.001
Laboratory results					
	Hemoglobin (g/L)	110.00 (81.75, 130.00)	114.00 (88.00, 131.00)	107.00 (76.00, 128.00)	<0.001
	WBC (10^9^/L)	13.68 (9.50, 18.90)	13.20 (9.80, 17.70)	14.30 (9.30, 20.20)	0.076
	Platelet (10^9^/L)	150.50 (96.00, 215.00)	159.00 (114.00, 220.00)	138.00 (82.00, 206.00)	<0.001
	Albumin (g/L)	32.40 (28.40, 36.40)	33.00 (29.50, 36.60)	31.50 (27.20, 36.30)	0.001
	ALT (U/L)	83.00 (39.00, 200.00)	80.00 (39.00, 164.00)	87.00 (40.00, 225.00)	0.160
	AST (U/L)	146.50 (63.00, 406.10)	130.00 (58.00, 313.00)	160.00 (76.00, 474.00)	<0.001
	Bilirubin (µmol/L)	16.40 (11.30, 25.33)	15.50 (10.97, 23.80)	17.25 (12.00, 27.60)	0.018
	Creatinine (µmol/L)	108.95 (75.38, 162.05)	93.00 (69.00, 133.00)	128.90 (87.00, 185.00)	<0.001
	BUN (mmol/L)	7.98 (5.82, 12.03)	7.30 (5.42, 10.32)	8.84 (6.33, 13.80)	<0.001
	Glucose (mmol/L)	9.11 (6.95, 12.28)	8.56 (6.69, 11.32)	9.60 (7.31, 13.01)	<0.001
	Sodium (mmol/L)	144.05 (140.60, 148.62)	143.00 (140.00, 146.70)	145.70 (141.50, 151.00)	<0.001
	Potassium (mmol/L)	3.94 (3.57, 4.40)	3.94 (3.60, 4.36)	3.94 (3.52, 4.44)	0.791
	Chlorine (mmol/L)	107.65 (103.90, 112.00)	107.20 (104.20, 111.00)	107.90 (103.30, 113.00)	0.272
	Calcium (mmol/L)	2.08 (1.97, 2.18)	2.07 (1.98, 2.17)	2.08 (1.96, 2.19)	0.855
	PT (s)	15.10 (13.70, 17.60)	14.70 (13.50, 16.40)	15.80 (14.00, 18.90)	<0.001
	INR	1.22 (1.09, 1.48)	1.18 (1.07, 1.35)	1.29 (1.12, 1.61)	<0.001
	Lactate (mmol/L)	2.90 (1.67, 6.22)	2.27 (1.43, 4.15)	3.80 (2.00, 8.30)	<0.001
	pH	7.39 (7.32, 7.45)	7.39 (7.34, 7.44)	7.37 (7.29, 7.45)	0.003
	PaO_2_ (mmHg)	128.85 (97.68, 165.48)	126.00 (98.25, 159.85)	132.00 (97.20, 170.00)	0.316
	PaCO_2_ (mmHg)	38.10 (32.50, 44.35)	38.90 (32.95, 43.75)	37.60 (32.00, 45.70)	0.677
	Actual bicarbonate (mmol/L)	22.90 (19.40, 26.10)	23.60 (20.40, 26.45)	22.20 (18.40, 25.60)	<0.001
	Base excess (mmol/L)	–2.08 (–5.80, 1.40)	–1.30 (–4.45, 1.75)	–2.90 (–7.20, 1.10)	<0.001
Scoring systems					
	SOFA	11.00 (9.00, 13.00)	9.00 (8.00, 11.00)	12.00 (10.00, 15.00)	<0.001
	Charlson Comorbidity Index	4.00 (2.00, 6.00)	4.00 (2.00, 5.00)	4.00 (2.00, 6.00)	0.001

Data are expressed as a number (n, %) or a median (interquartile range). ALT, alanine aminotransferase; AST, aspartate aminotransferase; BMI, body mass index; BUN, blood urea nitrogen; CRRT, continuous renal replacement therapy; DBP, diastolic blood pressure; ECMO, extracorporeal membrane oxygenation; IABP, intra-aortic balloon pump; INR, international normalized ratio; MBP, mean blood pressure; OHCA, out-hospital cardiac arrest; PCI, percutaneous coronary intervention; PT, prothrombin time; SBP, systolic blood pressure; SOFA, Sequential Organ Failure Assessment; WBC, white blood cell.

### 3.2 Variable Selection

**Fig. 2.**
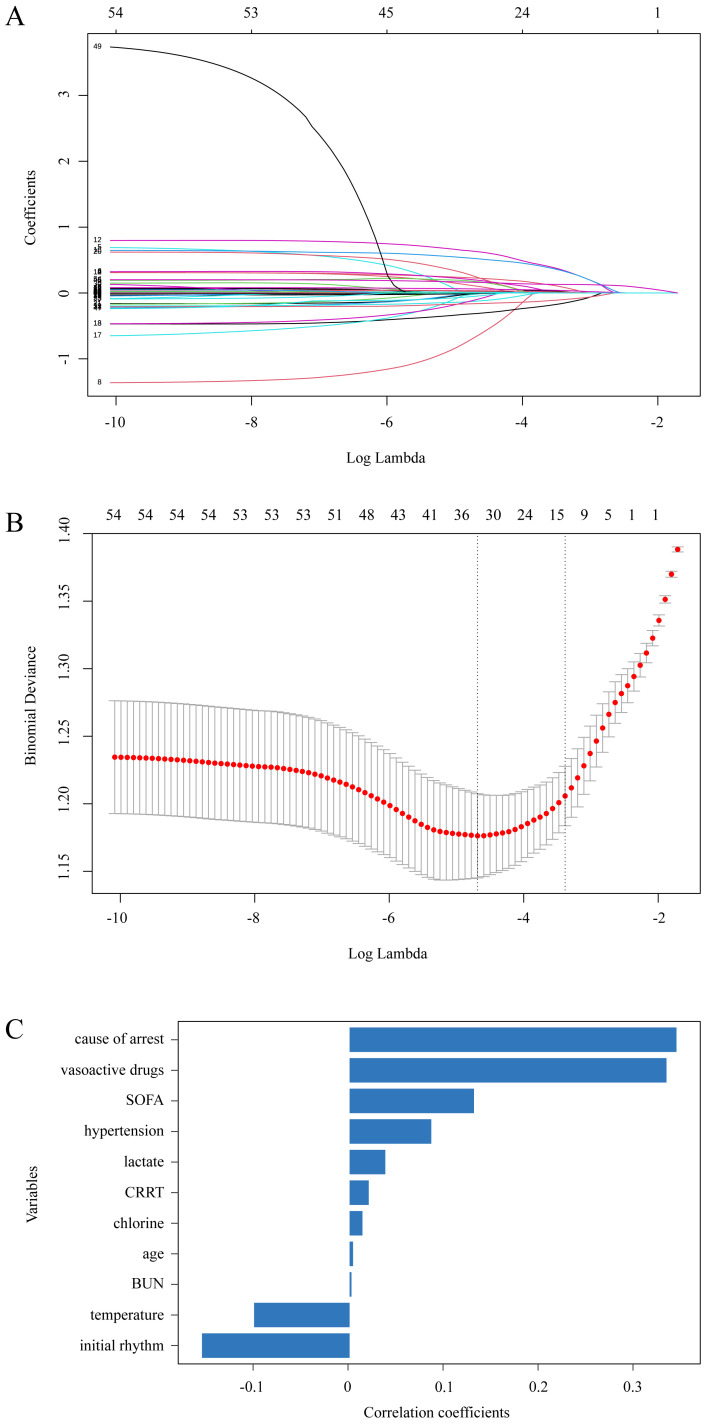
**Results of LASSO regression**. (A) Coefficient profile plot. (B) Cross validation plot. (C) Importance ranking of variables by regression coefficients.

**Fig. 3.**
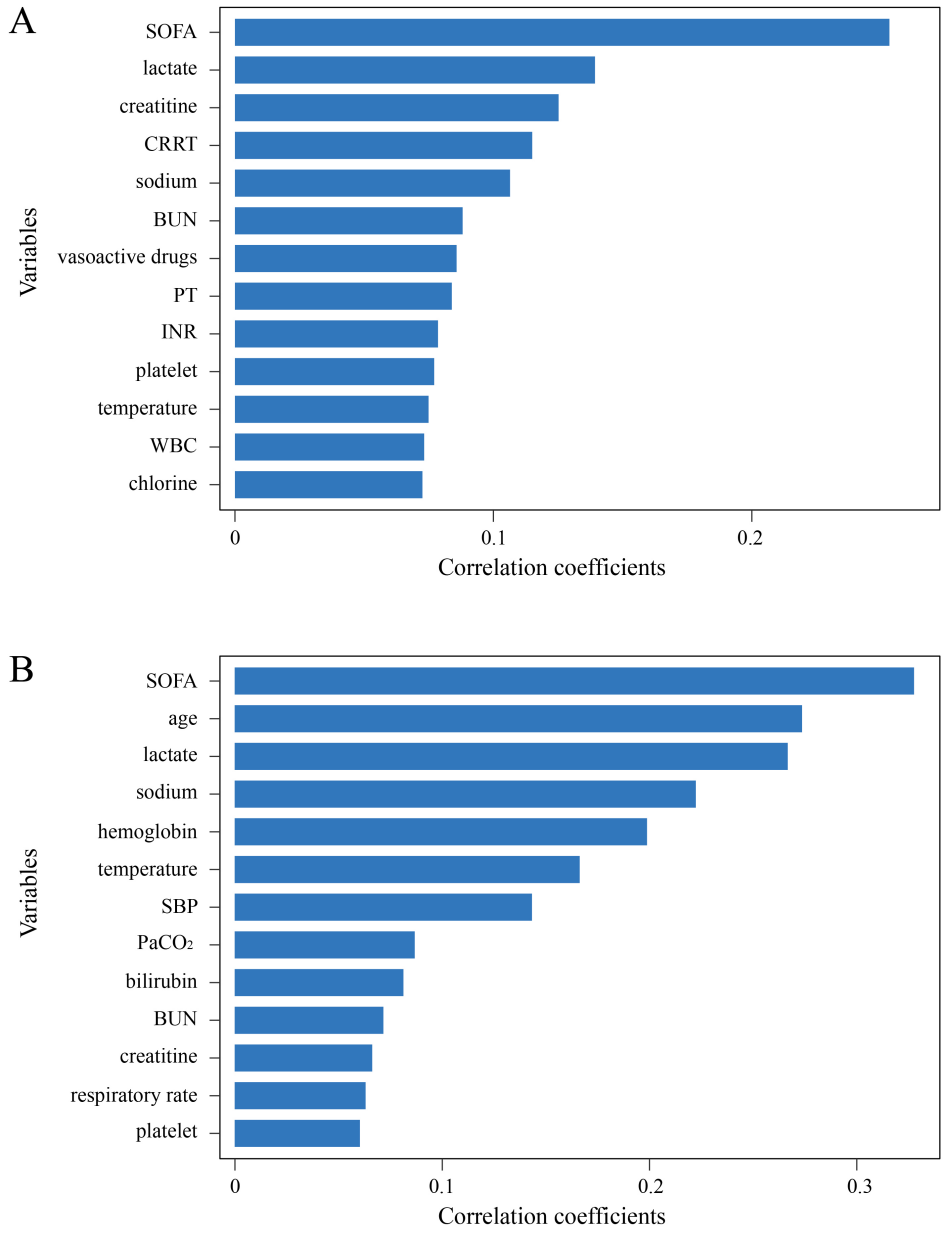
**Results of variable selection**. (A) Importance ranking of variables selected using RFE. (B) Importance ranking of variables selected using XGBoost.

Fig. 2A shows the coefficient profile plot for LASSO regression, wherein each curve represents a variable’s coefficient trajectory. As the log λ parameter increased, the regression coefficients continually converged toward zero. Fig. 2B shows the cross-validation plot for LASSO regression, with dotted lines indicating specific λ values. In particular, λmin on the left denotes the λ value with minimal likelihood deviation and an optimal model fit, retaining 33 variables, whereas λ-se on the right denotes one standard error from the minimum λ value, offering a good fit with a simpler model owing to the inclusion of fewer variables. Therefore, λ-se was chosen as the final criterion, which included 11 variables as follows: age, hypertension, non-cardiac cause of CA, shockable rhythm, vasoactive drugs, CRRT, temperature, chloride, BUN, lactate, and SOFA score. Fig. 2C shows the ranking of these variables by the magnitude of their regression coefficients. Similarly, RFE and XGBoost were used for variable selection, with Fig. 3A,B showing the top 13 variables.

Logistic regression analyses were used to identify risk factors for in-hospital mortality (Tables 2,3,4). The risk factors identified using LASSO regression included age, hypertension, non-cardiac cause of arrest, shockable rhythm, vasoactive drugs, CRRT, temperature, BUN, lactate, and SOFA. The risk factors identified using RFE included vasoactive drugs, CRRT, temperature, creatinine, BUN, sodium, lactate, and SOFA. In addition, the risk factors identified using XGBoost included age, temperature, respiratory rate, hemoglobin, bilirubin, creatinine, BUN, sodium, lactate, and SOFA.

**Table 2.** **Logistic regression analyses on variables selected using LASSO regression**.

Variable	Univariate			Multivariate		
	*p*-value	OR	95% CI	*p*-value	OR	95% CI
Age	0.036	1.01	1.01–1.02	0.039	1.02	1.00–1.04
Hypertension	0.002	1.90	1.28–2.83	0.030	1.68	1.05–2.69
Non-cardiac cause	0.002	1.68	1.20–2.35	<0.001	2.50	1.60–3.91
Shockable rhythm	0.011	0.62	0.43–0.89	0.002	0.77	0.40–0.82
Vasoactive drugs	<0.001	2.76	1.99–3.84	<0.001	2.47	1.64–3.70
CRRT	<0.001	2.71	1.94–3.78	0.032	1.26	1.03–2.05
Temperature	<0.001	0.67	0.57–0.79	0.010	0.79	0.65–0.94
BUN	<0.001	1.05	1.03–1.08	0.012	1.04	1.02–1.07
Chlorine	0.093	1.02	0.99–1.04	-	-	-
Lactate	<0.001	1.11	1.07–1.15	<0.001	1.07	1.04–1.14
SOFA	<0.001	1.20	1.14–1.26	0.004	1.09	1.03–1.16

CI, confidence interval; LASSO, Least Absolute Shrinkage and Selection Operator; OR, odds ratio.

**Table 3.** **Logistic regression analyses on variables selected using RFE**.

Variable	Univariate			Multivariate		
	*p*-value	OR	95% CI	*p*-value	OR	95% CI
Vasoactive drugs	<0.001	2.76	1.99–3.84	0.003	1.80	1.23–2.66
CRRT	<0.001	2.71	1.94–3.78	0.001	1.12	1.01–1.27
Temperature	<0.001	0.67	0.57–0.79	0.021	0.81	0.68–0.97
WBC	0.023	1.03	1.01–1.05	0.076	1.02	0.99–1.05
Platelet	<0.001	0.99	0.99–0.99	0.665	1.00	1.00–1.00
Creatinine	<0.001	1.03	1.01–1.07	0.004	1.02	1.00–1.05
BUN	<0.001	1.05	1.03–1.08	0.019	1.03	1.00–1.06
Sodium	<0.001	1.06	1.03–1.08	0.034	1.03	1.00–1.07
Chlorine	0.272	1.02	0.99–1.05	-	-	-
PT	0.044	1.02	1.00–1.04	0.267	1.07	0.95–1.20
INR	0.133	1.17	0.95–1.43	-	-	-
Lactate	<0.001	1.11	1.07–1.15	0.010	1.07	1.02–1.12
SOFA	<0.001	1.20	1.14–1.26	<0.001	1.16	1.08–1.24

RFE, recursive feature elimination.

**Table 4.** **Logistic regression analyses on variables selected using XGBoost**.

Variable	Univariate			Multivariate		
	*p*-value	OR	95% CI	*p*-value	OR	95% CI
Age	<0.001	1.01	1.01–1.02	0.002	1.02	1.01–1.03
SBP	0.150	1.00	0.99–1.00	-	-	-
Respiratory rate	0.020	1.05	1.01–1.07	0.041	1.04	1.00–1.07
Temperature	<0.001	0.67	0.59–0.77	0.004	0.80	0.69–0.93
Hemoglobin	0.046	0.99	0.99–0.99	0.021	1.01	1.01–1.01
Platelet	<0.001	0.99	0.99–0.99	0.764	1.00	1.00–1.00
Bilirubin	0.004	1.01	1.01–1.03	0.019	1.01	1.01–1.02
Creatinine	<0.001	1.01	1.00–1.01	0.003	1.01	1.01–1.01
BUN	<0.001	1.05	1.03–1.08	0.001	1.02	1.00–1.05
Sodium	<0.001	1.06	1.04–1.08	<0.001	1.05	1.03–1.08
PaCO_2_	0.227	1.01	0.99–1.02	-	-	-
Lactate	<0.001	1.12	1.09–1.16	0.003	1.06	1.02–1.10
SOFA	<0.001	1.25	1.19–1.30	<0.001	1.22	1.14–1.30

XGBoost, eXtremely Gradient Boosting.

### 3.3 Development and Validation of Models

**Fig. 4.**
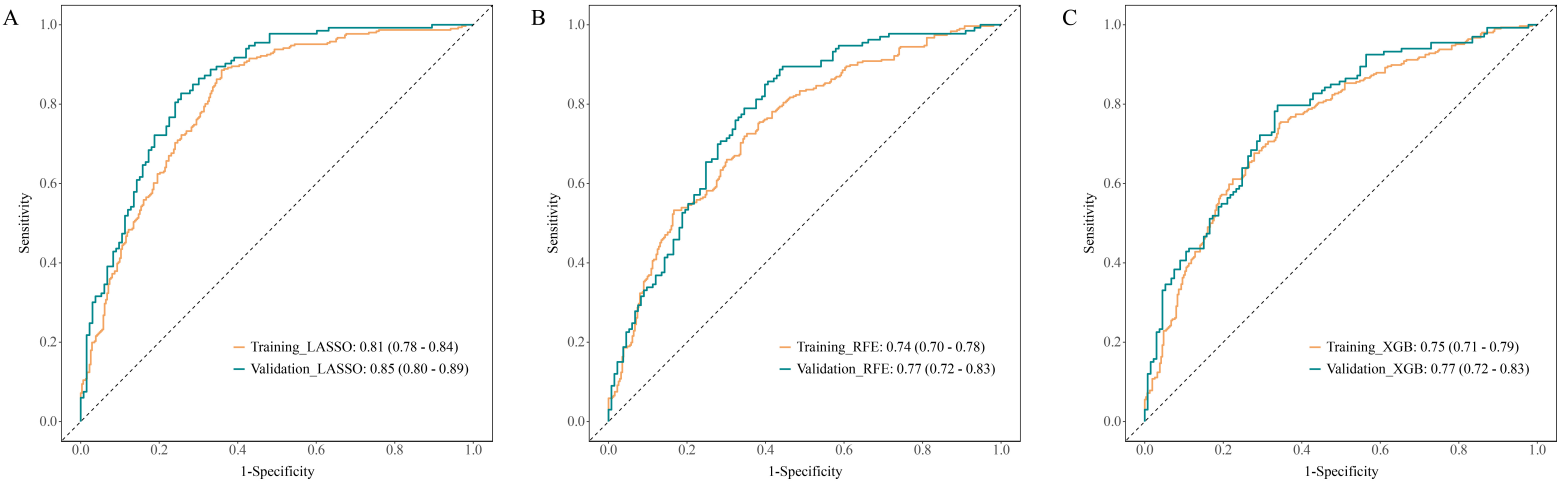
**ROC curves of prediction models constructed from variables selected using LASSO regression, RFE, and XGBoost in the training and internal validation sets**. (A) The LASSO model. (B) The RFE model. (C) The XGBoost model. ROC, receiver operating characteristic.

**Fig. 5.**
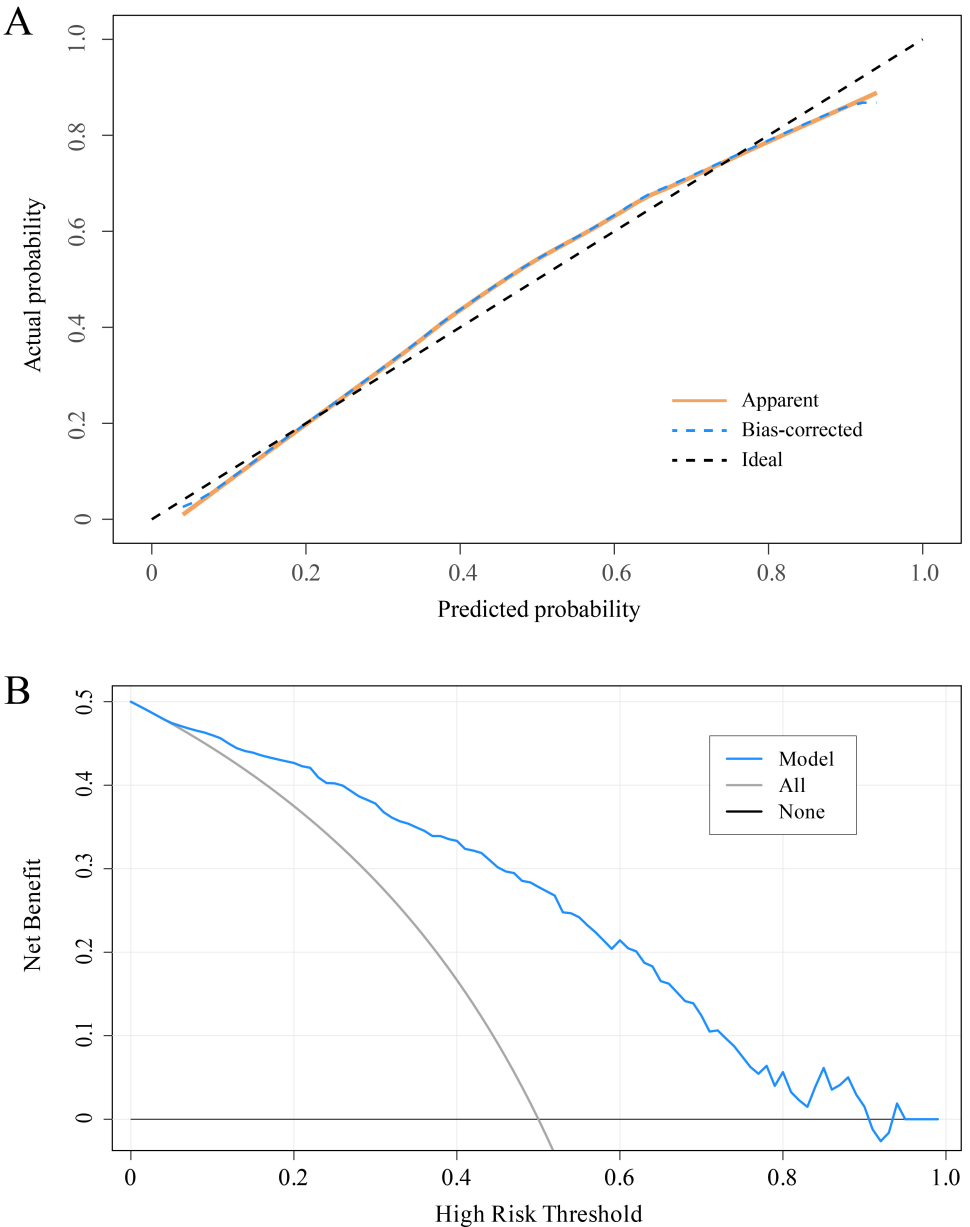
**Calibration curve and decision curve of the LASSO model**. (A) Calibration curve. (B) Decision curve.

Multivariate logistic regression was used to develop models for predicting the risk of in-hospital mortality in CA patients. As shown in Fig. 4A, the LASSO model demonstrated superior discriminative ability in both the training and internal validation sets, with AUC values of 0.81 (0.78, 0.84) and 0.85 (0.80, 0.89), specificity of 0.89 (0.85, 0.92) and 0.89 (0.83, 0.94), and sensitivity of 0.64 (0.58, 0.69) and 0.67 (0.59, 0.75), respectively. The AUC values of the RFE model were 0.74 (0.70, 0.78) and 0.77 (0.72, 0.83) and those of the XGBoost model were 0.75 (0.71, 0.79) and 0.77 (0.72, 0.83) in the training and internal validation sets, respectively (Fig. 4B,C). The calibration curve of the LASSO model exhibited high consistency between predicted and observed outcomes, and the decision curve confirmed the clinical utility of the model (Fig. 5).

Totally 204 eligible CA patients were included in the external validation, of which the in-hospital mortality was 51.47% (105/204). **Supplementary Table 1** showed that there were significant differences between the survivor and non-survivor groups for all 10 selected predictors (*p* < 0.05). The ROC curve was shown in **Supplementary Fig. 1**, with AUC value of 0.84 (0.78, 0.90), specificity of 0.74 (0.65, 0.83) and sensitivity of 0.82 (0.74, 0.90).

### 3.4 Presentation of the Nomogram

**Fig. 6.**
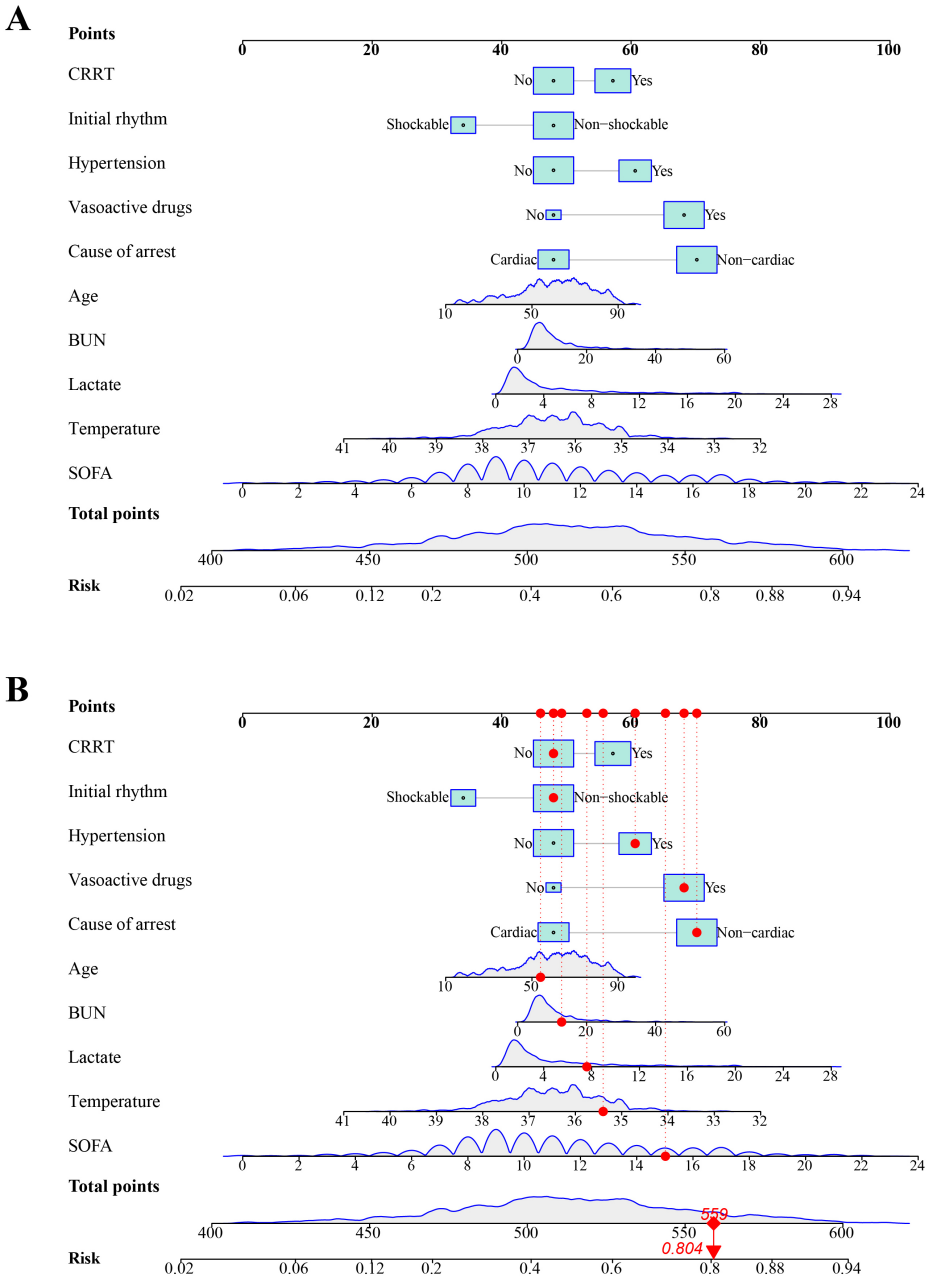
**Nomogram for predicting the risk of in-hospital mortality in CA patients**. (A) Nomogram. (B) An example of nomogram, with red dots for actual values and corresponding scores. The patient had a total score of 559 and a predicted risk of mortality of 0.804.

As the LASSO model exhibited optimal predictive performance, it was used to create a nomogram for predicting in-hospital mortality (Fig. 6A). Each variable in the nomogram was presented as a scaled line, with the length of the line indicating the impact of the variable on prediction. Specifically, longer lines indicated a greater contribution. The score of each variable was located on the “Points” row, and the total score corresponding to the probability of mortality was shown at the bottom, with higher scores indicating a higher risk of mortality risk. Fig. 6B showed an actual case. The specific variable values of this patient were marked in the nomogram to find the corresponding points, and the total points were calculated to be 559, with the corresponding mortality risk going to 0.804.

## 4. Discussion

In this retrospective study, a total of 996 CA patients were included and the efficacy of three methods in selecting variables for prediction models was compared. The LASSO regression approach revealed 10 independent risk factors, demonstrating a superior discriminative ability to predict in-hospital mortality. The results of external validation supported the good performance of the model. Consequently, the LASSO model was represented as a nomogram to improve its clinical applicability.

Redundant variables may introduce multicollinearity and lead to model overfitting, as well as increasing model complexity and reducing the interpretability and operability. Consequently, the process of variable selection is imperative, as it contributes to the creation of a model with both accuracy and efficiency. LASSO regression converges variables based on L1 regularization, reduces model overfitting and enhances its interpretability [21]. RFE offers high flexibility and shows compatibility with various algorithms, identifying the most impactful variable set through continuous iteration [22]. But the results of RFE can be compromised if the algorithm is insensitive to relevant variables [22]. XGBoost effectively quantifies the importance of variables in the ensemble tree models and captures non-linear relationships and variable interactions, showing strong robustness in the computation of large datasets [23]. However, both RFE and XGBoost are susceptible to overfitting, particularly in case of small sample sizes, high noise, or overly complex models. In addition, we found that XGBoost did not consider categorical variables, such as cause of CA, initial rhythm, and vasoactive drugs (Fig. 2C), potentially owing to the reduced accuracy of feature importance after one-hot encoding [23]. Therefore, LASSO regression shows strong potential in screening key features [24, 25]. In this study, the LASSO model had superior discriminative ability and adaptability, which highlighted its advantages in variable selection.

Assessing the prognosis of CA patients has consistently been a subject that requires the attention and resolution for clinicians. Current research has raised a spectrum of evaluation methods, mainly encompassing risk scores derived from multiple regression analysis [26, 27, 28] and prediction models based on ML algorithms [29, 30, 31]. ML-based models have demonstrated promising predictive capabilities; however, nomogram has certain valuable advantages when used for survival analyses, especially for interpreting the outputs. Nomogram facilitates interaction with clinicians through a user-friendly, operable interface. It integrates multidimensional variables into a single chart, simplifying complex calculations and rendering the results intuitive and easy to comprehend. Fig. 6B displayed the actual prediction process for a patient. By merely collecting the values of the required variables, the risk of in-hospital mortality can be intuitively obtained through the nomogram, thereby enabling a personalized assessment of patient prognosis. Consequently, nomograms have been extensively employed in research as an advantageous clinical prediction model.

Chen *et al*. [32] and Sun *et al*. [33] both extracted data from the Medical Information Mart for Intensive Care IV (MIMIC-IV) database to construct nomogram models for predicting the risk of in-hospital mortality in CA patients, with AUC values of 0.801 (0.775, 0.835) and 0.799 (0.762, 0.837) in internal validation, respectively. Both studies used LASSO regression and logistic regression to identify independent risk factors. Chen *et al*. [32] selected age, malignant cancer, norepinephrine, heart rate, respiratory rate, temperature, peripheral oxygen saturation (SpO2), sodium, BUN, bicarbonate, and lactate, while Sun *et al*. [33] chose age, gender, heart rate, mean arterial pressure (MAP), respiratory rate, temperature, SpO2, PT, bicarbonate, Glasgow Coma Scale (GCS) score, and SAPS III score. However, considering the differences in the characteristics of the cohorts, the models based on the MIMIC-IV database often exhibit significant limitations in generalizability, with these two studies lacking the support from external validation. Nagy *et al*. [34] developed a nomogram for predicting 30-day mortality in 103 Out-of-hospital Cardiac Arrest (OHCA) patients, with the AUC value of 0.835 (0.755, 0.907) in internal validation. Variable selection was achieved through restricted cubic splines and the Akaike information criterion, including age, initial rhythm, heart rate, pH, and right ventricular end-diastolic diameter. Zhang *et al*. [18] developed a nomogram for predicting in-hospital mortality in 561 in-hospital cardiac arrest (IHCA) patients, identifying only rearrest, duration of cardiopulmonary resuscitation (CPR), and length of hospital stay as three independent risk factors through multivariate logistic regression, yet demonstrating good discriminative ability with an AUC value of 0.88 (0.83, 0.93). Both studies constructed robust nomogram models with fewer predictive factors, but faced issues with small sample sizes and a lack of external validation.

In this retrospective study, we initially constructed a nomogram model for predicting in-hospital mortality risk in a relatively large cohort of CA patients with an AUC value of 0.85 (0.80, 0.89). The calibration and decision curve confirmed its clinical applicability. Furthermore, we substantiated the generalizability of the model in the external validation set, with an AUC value of 0.84 (0.78, 0.90). Variable selection was achieved through LASSO regression and multivariate logistic regression, identifying age, hypertension, initial rhythm, cause of CA, CRRT, vasoactive drugs, temperature, BUN, lactate, and SOFA score as independently associated with mortality. These variables are readily accessible in clinical settings, enhancing the practicability and operation convenience of the nomogram.

However, this study has a few limitations that should be acknowledged. First, its retrospective nature might have introduced unavoidable biases. Second, although the final model was validated in an external cohort, the sample size is small and further validation in large-scale cohort is required. Third, OHCA and IHCA were simultaneously included in the analysis; consequently, some significant independent risk factors specifically associated with either of these conditions might have been overlooked.

## 5. Conclusions

In this study, we used variables identified via LASSO regression to developed a nomogram for predicting the in-hospital mortality in CA patients. The nomogram demonstrated robust discriminative ability and clinical utility.

## Availability of Data and Materials

The datasets supporting the conclusions of this article are available from the corresponding author on reasonable request.

## Supplementary Material

Supplementary material associated with this article can be found, in the online version, at https://doi.org/10.31083/RCM33387.
