# Supplementary material for: Development and Validation of a Nomogram Prediction Model for In-hospital Mortality in Patients with Cardiac Arrest: A Retrospective Study
Source: Rev Cardiovasc Med. 2025 Apr 10;26(4):33387. doi: 10.31083/RCM33387 (PMC12059783; doi:10.31083/RCM33387)
Supplement: Supplementary file 1 [file 2153-8174-26-4-33387-s1.docx]

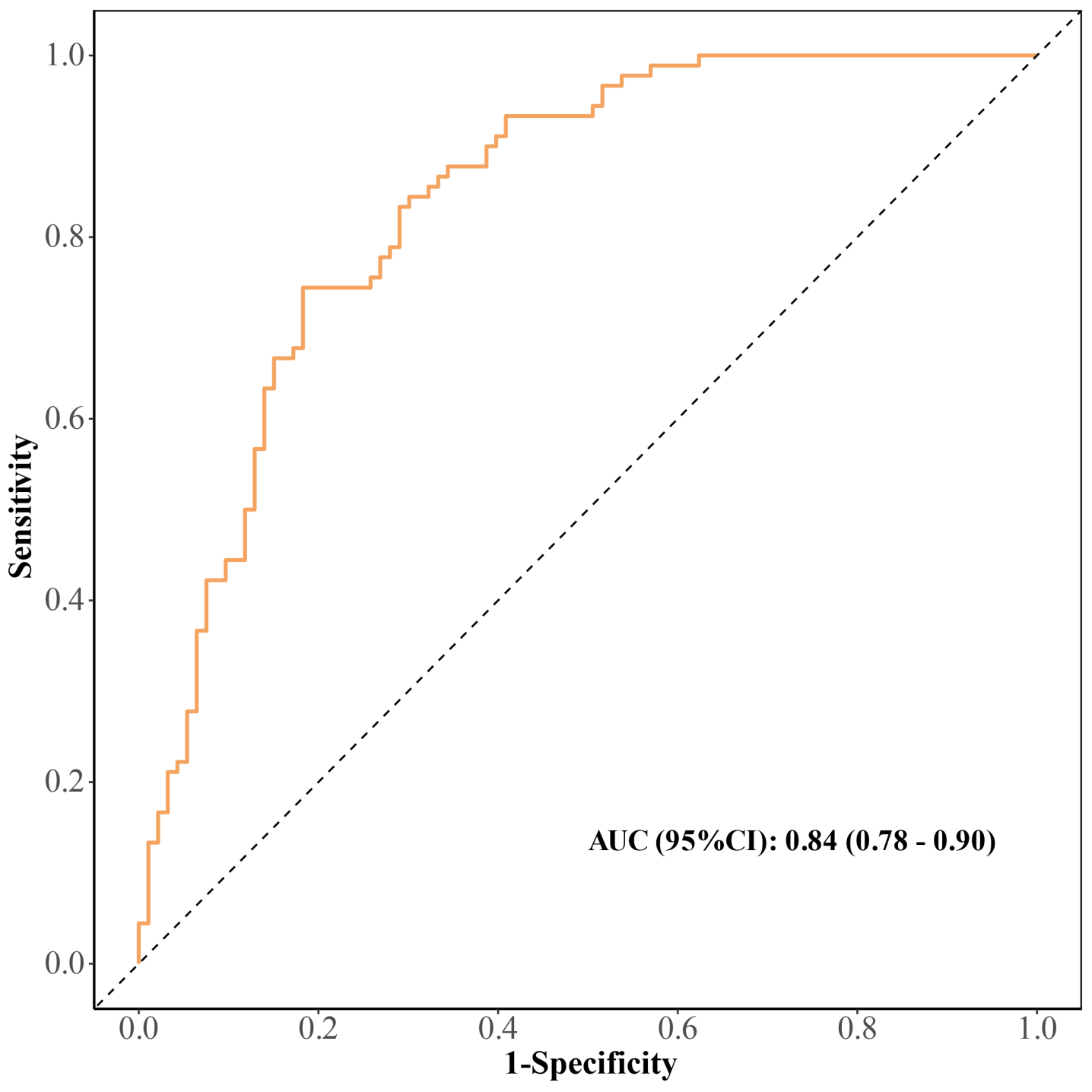


**Supplementary Fig. 1. External validation of the LASSO model.**

| **Variables** | **Overall (n = 204)** | **Survivor (n = 99)** | **Non-survivor (n = 105)** | ***p*-value** |
| --- | --- | --- | --- | --- |
| Age | 63.50 (49.00, 73.00) | 62.00 (49.50, 72.00) | 66.00 (49.00, 74.00) | 0.068 |
| Temperature | 36.50 (35.80, 37.20) | 36.70 (35.85, 37.30) | 36.20 (35.80, 37.00) | 0.042 |
| BUN | 7.92 (5.75, 11.36) | 7.20 (5.54, 10.64) | 8.70 (6.43, 11.90) | 0.042 |
| Lactate | 4.20 (1.98, 5.20) | 3.00 (1.70, 4.40) | 4.96 (3.23, 6.80) | <.001 |
| SOFA | 10.00 (9.00, 12.00) | 9.00 (9.00, 10.50) | 12.00 (10.00, 13.00) | <.001 |
| Hypertension | 86 (42.16) | 37 (37.37) | 49 (51.04) | 0.033 |
| Non-cardiac causes | 126 (61.76) | 52 (41.27) | 74 (58.73) | 0.008 |
| Shockable rhythm | 64 (31.37) | 37 (57.81) | 27 (42.19) | 0.043 |
| Vasoactive drugs | 186 (91.18) | 86 (46.24) | 100 (53.76) | 0.035 |
| CRRT, n(%) | 68 (33.33) | 24 (35.29) | 44 (64.71) | 0.007 |

**Supplementary Table 1. Baseline characteristics for external validation set.**
